# Short-Term Fasting Induces Hepatocytes’ Stress Response and Increases Their Resilience

**DOI:** 10.3390/ijms26030999

**Published:** 2025-01-24

**Authors:** Patrik Prša, Izak Patrik Miller, Barbara Kramar, Dušan Šuput, Irina Milisav

**Affiliations:** 1 Institute of Pathophysiology, Faculty of Medicine, University of Ljubljana, Zaloska 4, SI-1000 Ljubljana, Slovenia; 2 Laboratory of Oxidative Stress Research, Faculty of Health Sciences, University of Ljubljana, Zdravstvena pot 5, SI-1000 Ljubljana, Slovenia

**Keywords:** hormesis, time-restricted feeding, apoptosis, caspase-9, oxidative stress, hydrogen peroxide

## Abstract

Fasting leads to a range of metabolic adaptations that have developed through evolution, as humans and other mammals have unequal access to food over the circadian cycle and are therefore adapted to fasting and feeding cycles. We have investigated the role of a single fasting episode in rats in triggering the stress response of liver hepatocytes. Since the stress responses were observed in both animals and isolated cells, we investigated whether the effects of the animal stressor could persist in the cells after isolation. By measuring staurosporine-induced apoptosis, stress signalling, and oxidative and antioxidant responses in hepatocytes from fasted and ad libitum-fed animals, we found that only fasting animals elicited a stress response that prevented caspase-9 activation and persisted in isolated cells. The addition of glucose oxidase, a hydrogen peroxide-producing enzyme, to the cells from ad libitum-fed animals also led to a stress response phenotype and prevented the activation of caspase-9. A single fasting episode thus leads to a stress response in normal hepatocytes, with hydrogen peroxide as a second messenger that reduces the initiation of apoptosis. This finding is the first characterisation of a mechanism underlying the effects of fasting and provides a basis for the development of methods to increase the resilience of cells. These findings need to be taken into account when interpreting the results obtained in animal and cell research models to account for the effects of overnight fasting used in many laboratory protocols. The research results also form the basis for the development of clinical applications to increase the resistance of transplants and to improve the fitness of hepatocytes under acute stress conditions in liver and some metabolic diseases.

## 1. Introduction

Interest in fasting research, especially intermittent fasting (IF), has increased in the last few years. Metabolic improvements and life extension have been reported in IF and calorie restriction experiments in various animal species, including humans [1]. As unrestricted amounts of food can be consumed in non-fasting periods during IF, this technique could increase compliance compared to calorie restriction. The term IF includes different modalities, the most common of which are (1) alternate-day fasting, (2) fasting on pre-determined weekdays, and (3) time-restricted feeding (TRF), in which food consumption is restricted to certain hours of the day followed by a 10–16-h fasting period [1,2]. Different gene expression signatures were found between calorie restriction and TRF [3,4]; nevertheless, some effects attributed to calorie restriction may be due to TRF [1]. Namely, in calorie restriction experiments, food is often given at a fixed time of day, which rodents and higher animals consume within a few hours in a pattern reminiscent of TRF [3]. Research methods often involve the TRF/fasting of laboratory animals. Examples include studies on the effects of insulin, observing toxicological effects in pathological assessments by autopsy, and improving histological studies of the liver [5]. Laboratory protocols for the isolation of mitochondria from rat liver are also based on overnight fasting to reduce glycogen and isolate viable mitochondria [6,7].

Intermittent fasting elicits adaptive cellular responses that increase maintenance and repair, stimulate mitochondrial biogenesis, promote cell survival, minimize growth and reproduction, and enhance stress resistance [1]. An adaptive stress response to IF can increase the transcription of stress-induced proteins, such as heat shock protein (HSP) 70 [8,9], and increase the expression of antioxidant defences, e.g., by upregulating nuclear factor erythroid 2-related factor 2 (Nrf2) and superoxide dismutase (SOD2) [10]. It can also result in increased total antioxidant capacity [10], DNA repair, protein quality control, mitochondrial biogenesis, and autophagy [11], and the downregulation of inflammation [1]. These processes support health improvements and resistance to diseases.

The induction of many antioxidative defence mechanisms by IF indicates an important role of reactive oxygen species (ROS) in stress response signalling. This is consistent with the notion that moderate concentrations of ROS are required for normal cell function, and that an excessive or insufficient ROS leads to oxidative imbalance, i.e., oxidative stress or antioxidative/reductive stress, respectively [12]. Hydrogen peroxide (H_2_O_2_), a ROS molecule, serves as the main redox-sensing and signalling molecule and is reduced to water by the antioxidant enzymes glutathione peroxidases (GPx) and catalase (CAT) [13,14,15].

Dysregulation of ROS and other triggers can induce apoptosis in liver hepatocytes [16]. In general, apoptosis induced by external stimuli is propagated through FAS/CD95 receptors that result in the activation of caspase-8 [17]. However, in type II cells, such as hepatocytes, the signals from caspase-8 must be amplified by the intrinsic (mitochondrial) signalling pathway, which involves the activation of caspase-9, to efficiently trigger apoptosis [18]. The protein BH3-interacting domain death agonist (Bid) belongs to the B cell chronic lymphocytic leukemia/lymphoma protein (BCL-2) family and can convey the signals between these two pathways, as it is cleaved by caspase-8 into a truncated form, tBid. tBid moves to the outer mitochondrial membrane to interact with the BCL-2 antiapoptotic family members Bcl-2; B-cell lymphoma, long isoform (Bcl-xL); and myeloid cell leukemia-1 (Mcl-1). tBid possibly even acts similarly to the proapoptotic pore-forming BCL-2 associated X protein (Bax) and BCL-2 antagonist killer (Bak) [19,20]. Commitment to apoptosis through the intrinsic apoptosis pathway largely depends on the relative amounts and interactions of pro- and antiapoptotic BCL-2 proteins. The best-known proapoptotic members are Bak in the mitochondrial outer membrane and cytosolic Bax, which when activated, form pores in the outer mitochondrial membrane and/or enable the release of proteins such as cytochrome c and second mitochondria-derived activator of caspase (Smac/Diablo) [21,22]. Cytochrome c is a part of the complex that activates caspase-9, while Diablo counterbalances inhibitor of apoptosis (IAP) proteins [19]. Complex interactions between pro- and antiapoptotic proteins therefore prevent unnecessary apoptosis and, conversely, enable efficient apoptosis initiation through the main initiator caspase of an intrinsic apoptosis pathway, caspase-9. This caspase is, therefore, activated by external and internal signals in hepatocytes and conveys signals to the execution caspases-3/7 for efficient apoptosis triggering.

Moderate stress can induce cell stress responses, rather than cell death, resulting in enhanced defence, repair, and cross-resistance to multiple stressors [21,23]. The preapoptotic cell stress response (PACOS) is a stress adaptation of primary hepatocytes, which reduces apoptosis initiation through the inactivation of caspase-9 that would otherwise be activated by a moderate stressor [24,25]. Severe stress can still activate apoptosis through the activation of caspase-3/7 independent of caspase-9. The PACOS is triggered by a moderate increase in H_2_O_2_ [26].

Since stress responses have been measured in animals and isolated cells, we investigated whether the effects of the animal stressor can persist in the cells after isolation. Indeed, a single 17-h fast in rats induces a stress response (PACOS) in isolated hepatocytes. We show that larger amounts of H_2_O_2_ are formed in the hepatocytes of fasted compared to ad libitum-fed animals upon isolation, while there are lower antioxidant enzyme activities. Gene expression patterns differ between the two states, with the antiapoptotic gene *Mcl1* highly overexpressed a day after fasting, as are the genes for the antioxidant enzymes and stress response proteins. We have confirmed that H_2_O_2_ is a crucial signalling molecule that enables the transition from the normal to the stress-response state. Thus, we report a mechanism underlying fasting that increases the resilience of liver cells.

## 2. Results

### 2.1. Cell Damage and Apoptosis

**Figure 1:**
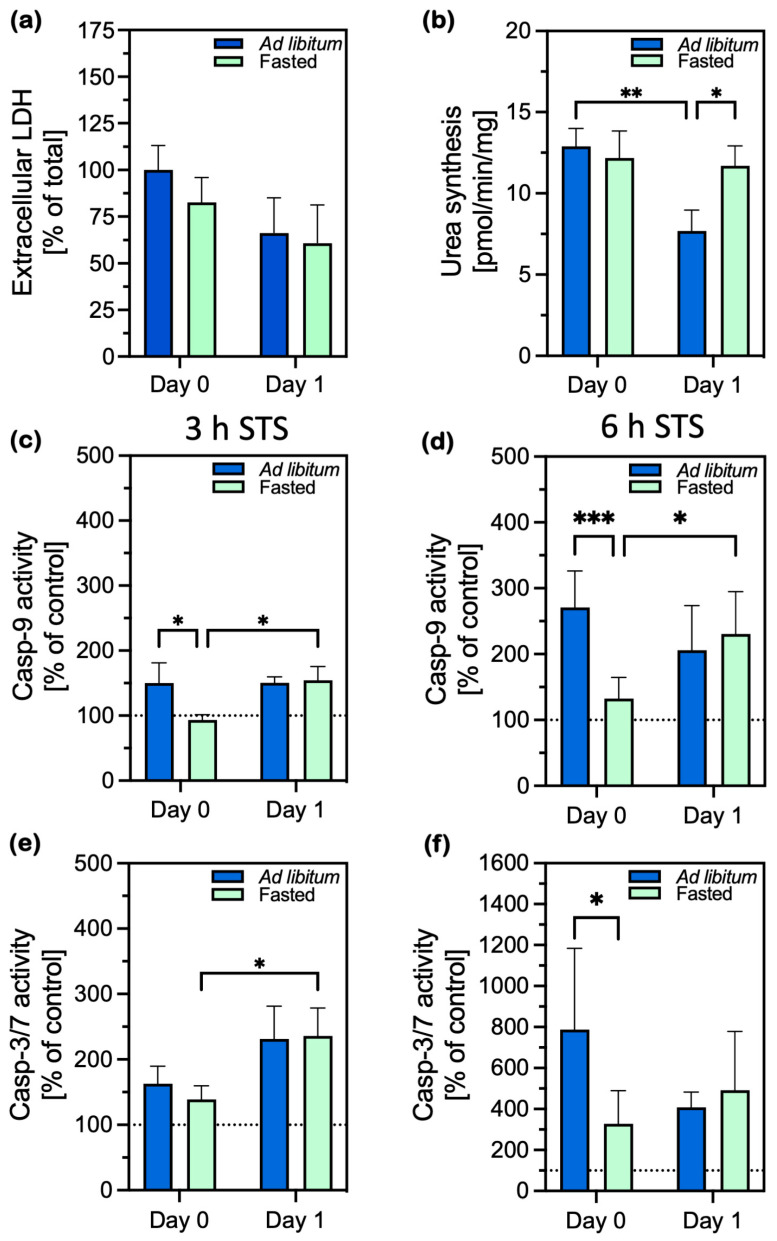
Hepatocyte damage, function, and caspase activity in hepatocytes immediately (day 0) and 24 h (day 1) after isolation from ad libitum-fed (blue bars) and fasted (green bars) animals. (**a**) Release of lactate dehydrogenase (LDH) into extracellular medium, calculated as the average percentage of LDH released from the cells from fed animals; (**b**) urea synthesis in the presence of ammonium chloride; (**c**,**d**) caspase-9 activation induced by staurosporine (STS) treatment for 3 and 6 h, respectively, expressed as percentages of the untreated control; caspase activity of untreated controls is indicated by a dotted line. (**e**,**f**) Caspase-3 activation induced by staurosporine (STS) treatment for 3 and 6 h, respectively, expressed as percentages of the untreated control. Caspase activity of untreated controls is indicated by a dotted line. All the data are presented as the mean ± standard deviation (SD) and were analyzed with two-way ANOVA, followed by Tukey’s test to test for differences between samples. (**a**) *n* = 4; (**b**,**c**,**e**) *n* = 3; (**d**,**f**) *n* = 7. Casp: caspase; * *p* ≤ 0.05, ** *p* ≤ 0.01, *** *p* ≤ 0.001. For the exact *p* values, please refer to Table S1.

First, we looked at whether animal fasting affected cell integrity and measured the release of lactate dehydrogenase (LDH) into the surrounding medium. Similar LDH release from both types of cells—those from the ad libitum-fed and fasted animals—was observed. Differences in the extracellular release of LDH were small and not statistically significant on either day 0 or 1 (Figure 1a). These data are supported by a constant survival rate of the cells of the fasted animals, which was determined using the MTT test (Supplementary Figure S1a).

Ammonia removal through urea production is among the key functions of hepatocytes and is a good indicator of hepatocyte functionality and mitochondrial integrity. Urea production was equal in both groups of cells on the day of isolation. A day later, significantly less urea was produced only in the cells of the fed animals, while the cells of the fasted animals produced just as much urea as on the isolation day (Figure 1b). As we measured urea synthesis by cells stimulated with ammonia, these measurements indicate the hepatocytes’ ability for urea synthesis rather than protein turnover. Therefore, the results of the urea measurement indicate that all the isolated cells could produce urea. However, a day after isolation, only the cells of fasted animals retained the ability to produce just as much urea as on the isolation day, implying that the cells from the fasted animals functioned better on day 1.

We further compared the effects on apoptosis induction when the two types of cells were treated with staurosporine (STS), a potent activator of caspase-9. Overnight fasting completely prevented the activation of caspase-9 after 3 h of STS treatment on day 0, with the caspase-9 activity being equal to that of the STS-untreated control (Figure 1c). In contrast, caspase-9 increased significantly in the cells of fed animals, to 150% of that of the untreated control, which was about 50% more than in the cells of fasted animals. Caspase-9 was induced equally one day after isolation in both groups of cells. The pattern described was also observed 6 h after STS-induced apoptosis. Here too, caspase-9 was statistically significantly activated in the cells of the fed animals, twice as strongly as in the cells of the fasted animals (Figure 1d).

The lower activation of caspase-9 on the day of isolation was reflected in lower activation of caspase-3/7 in cells isolated from fasted animals, which was statistically significant after 6 h of STS induction (Figure 1f).

**Figure 2:**
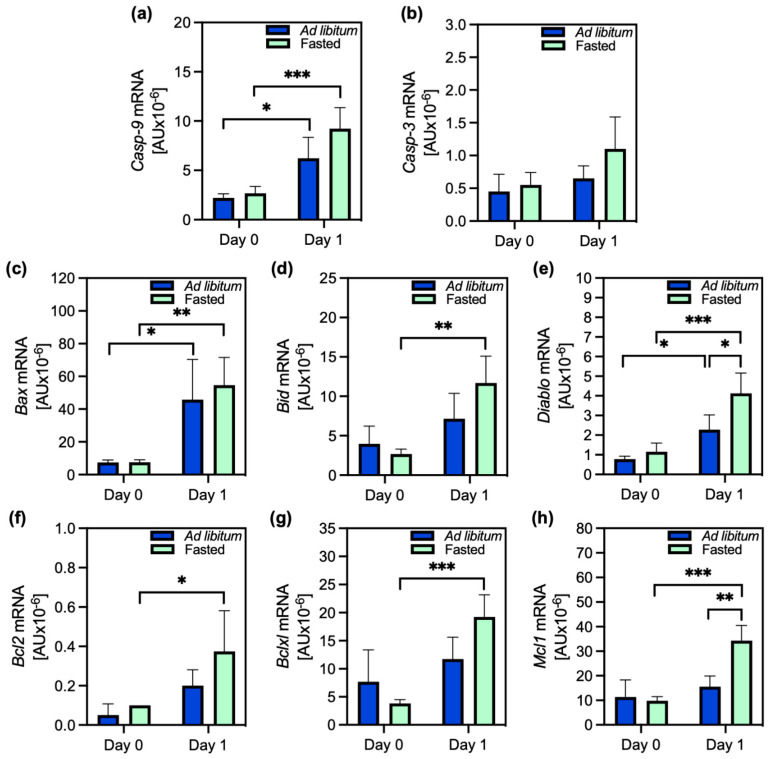
Expression of apoptotic and antiapoptotic genes. Gene expression of (**a**) caspase-9 (*Casp9*), (**b**) caspase-3 (*Casp3*), (**c**) *Bax*, (**d**) *Bid*, (**e**) *Diablo*, (**f**) *Bcl2*, (**g**) *Bclxl*, and (**h**) *Mcl1* in the hepatocytes of ad libitum-fed (blue bars) and fasted (green bars) animals, collected at 0 (day 0) and 24 h (day 1) after cell isolation. Data are presented as the mean ± SD and were analyzed by two-way ANOVA, followed by Tukey’s test, to test for differences between samples. (**a**–**h**): *n* = 4. * *p* ≤ 0.05, ** *p* ≤ 0.01, *** *p* ≤ 0.001. For the exact *p* values, please refer to Table S2.

The caspase-9 gene was equally expressed in the cells of fed and fasted animals on the isolation day and overexpressed in both a day after isolation (day 1, Figure 2a). Statistical significance was reached only within the same group of cells, i.e., either from fasted or fed animals. A similar expression pattern was observed for caspase-3, though about 10× less mRNA was produced, and there were no statistically significant differences among the groups (Figure 2b). There was a lower expression of BCL-2 family genes in the cells of both fasted and fed animals on the isolation day compared to the day after, a pattern similar to the one observed for caspase expression. Bcl2 was 10–100 times less expressed than other pro- and anti-apoptotic genes according to the mRNA/cDNA content (Figure 2). Of the proapoptotic members, Bax was most strongly expressed, and its expression was not statistically different between the cells from fed and fasted animals. Anti-apoptotic Bclxl had a similar expression pattern. Similar amounts of mRNA for Bax were also produced for another anti-apoptotic protein Mcl-1; however, in contrast to *Bax*, *Mcl1* was highly significantly overexpressed in the cells from fasted animals one day after isolation, compared to the cells from ad libitum-fed animals (Figure 2h).

The expression of other pro-and anti-apoptotic members, except for the pro-apoptotic Diablo did not differ between the cells from fasted and fed animals. *Diablo* was significantly overexpressed in the cells from the fasted animals compared to the fed animals on day 1, but more than 8 times less mRNA was produced than that of *Mcl1*. In conclusion, the expression of all BCL-2 members significantly increased on day 1. The highest expression of *Mcl1* in the cells from fasted animals may contribute to the survival ability of these cells. Compared to the cells from ad libitum-fed animals, fasting prevented the activation of caspase-9 upon the triggering of apoptosis with STS, and consequently resulted in a lower activation of caspase-3 than in the presence of an active caspase-9, which can also be seen when comparing the 6-h STS activation in the cells from ad libitum and fasted animals (Figure 1d,f). Thus, fasting triggered a stress adaptation previously described as PACOS.

### 2.2. Oxidants and Antioxidants

**Figure 3:**
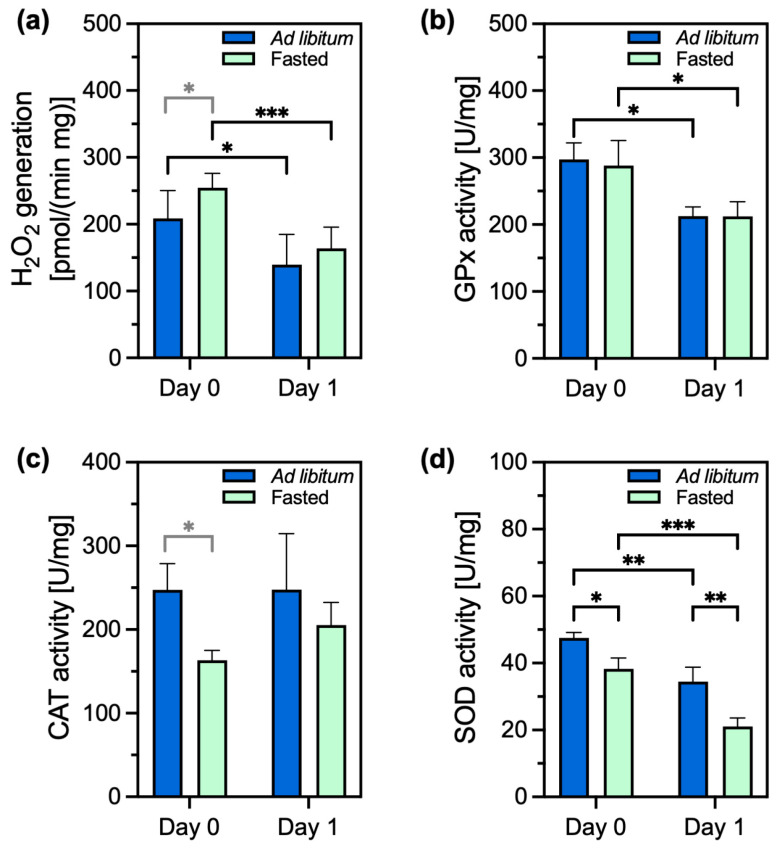
H_2_O_2_ production and antioxidant enzyme activities in primary hepatocytes of ad libitum-fed (blue bars) and fasted (green bars) animals at 0 (day 0) or 24 h (day 1) after hepatocyte isolation. (**a**) H_2_O_2_ production (*n* = 6); (**b**) glutathione peroxidase (GPx) activity (*n* = 3); (**c**) catalase (CAT) activity (*n* = 3); (**d**) superoxide dismutase (SOD) activity (*n* = 3). Enzyme activities are expressed per total protein in the sample. Data are presented as the mean ± standard deviation (SD) and were analyzed using two-way ANOVA, followed by Tukey’s test, to test for differences between samples. An unpaired *t*-test was used to compare the individual data sets and the results are shown in grey. * *p* ≤ 0.05, ** *p* ≤ 0.01, *** *p* ≤ 0.001. For the exact *p* values, please refer to Table S1.

The induction of the PACOS is mediated by an increase in reactive oxygen species. Therefore, we next assessed the oxidant and antioxidant status. The cells of fasted animals produced on average 1.2 times more H_2_O_2_ than those of fed animals on the day of isolation; the difference between the groups was statistically significant (Figure 3a). The amount of produced H_2_O_2_ was large on the day of isolation and significantly decreased the day after, reaching approximately equal amounts across the two groups of cells. The activity of glutathione peroxidase was significantly higher in both types of cells on the day of isolation than on day 1. GPx activity did not differ between the cells of fasted and ad libitum-fed animals (Figure 3b). The CAT (another H_2_O_2_-metabolizing enzyme) activity was on average 1.5 times higher in the cells of ad libitum-fed animals compared to that in fasted animals on the isolation day. The difference between the two groups was significant (Figure 3c). On day 1, when the amount of peroxide decreased, there was no significant difference between the cells from fed and fasted animals.

The activity of SOD was significantly higher in the cells of fed animals on the day of isolation than in the cells of fasted animals. SOD activity was reduced a day after isolation in both groups of cells, with the highest decrease in the cells of fasted animals. There was still significantly higher SOD activity in the cells of fed animals on day 1 (Figure 3d). In conclusion, more H_2_O_2_ was formed in the cells of fasted animals on the isolation day, while the activity of the antioxidant enzymes was equal to or lower than in the cells of the ad libitum-fed animals.

**Figure 4:**
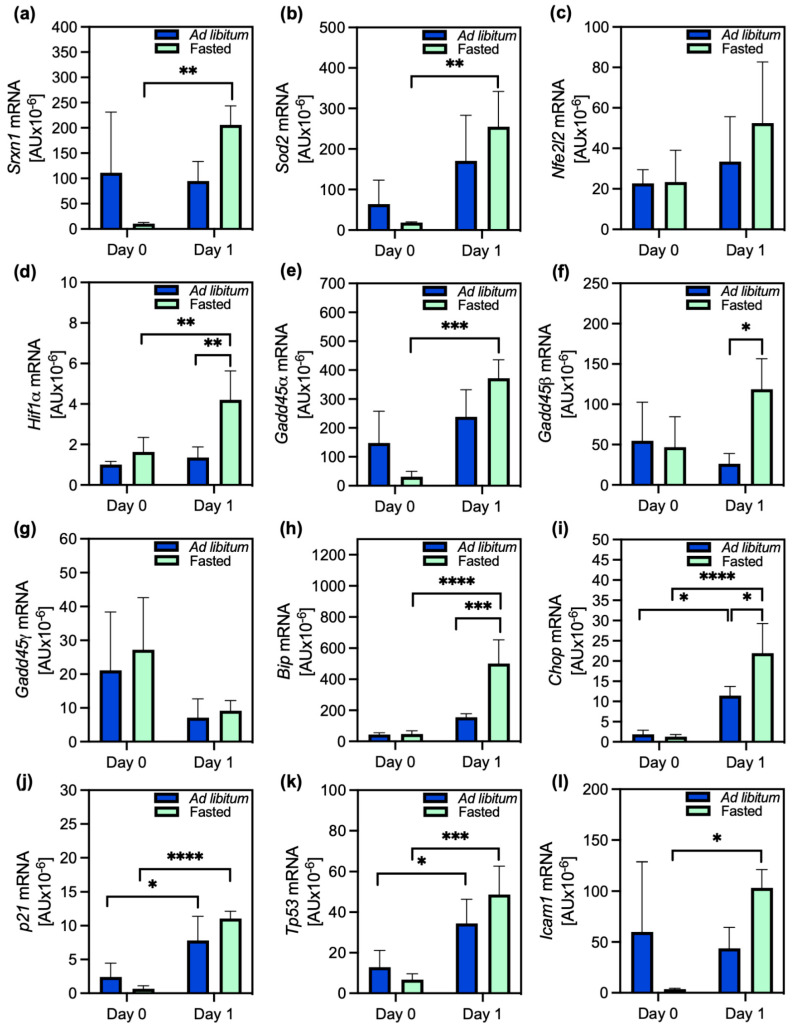
Expression of antioxidant, stress response, and inflammation-related genes. Gene expression of (**a**) sulfiredoxin-1 (*Srxn1*), (**b**) *Sod2*, (**c**) Nrf2 (*Nfe2l2*), (**d**) hypoxia-inducible factor-1α (*Hif1α*), (**e**) growth arrest and DNA damage-inducible 45 (*Gadd45α*), (**f**) *Gadd45β*, (**g**) *Gadd45γ*, (**h**) binding immunoglobulin protein (*Bip*), (**i**) C/EBP homologous protein (*Chop*), (**j**) protein p21 (*p21*), (**k**) tumour protein 53 (*Tp53*), and (**l**) inflammation: intercellular adhesion molecule 1 (*Icam1*) from primary hepatocytes of ad libitum-fed (blue bars) and fasted (green bars) animals collected at 0 (day 0) or 24 h (day 1) after cell isolation. Data are presented as the mean ± SD and were analyzed by two-way ANOVA, followed by Tukey’s test, to compare samples among each other. (**a**–**l**): *n* = 4; * *p* ≤ 0.05, ** *p* ≤ 0.01, *** *p* ≤ 0.001, **** *p* ≤ 0.0001. For the exact *p* values, please refer to Table S2.

The expression of the representative genes for the oxidative stress response—sulfiredoxin-1 (*Srxn1*), SOD2 (*Sod2*), Nrf2 (*Nfe2l2*), and hypoxia-inducible factor-1α (*Hif1α*)—also differed between the two groups of cells (Figure 4a–d). While *Srxn1* and *Sod2* were overexpressed in the cells of ad libitum-fed animals compared to the cells of the fasted animals on the day of isolation, there was little difference in the expression of the genes encoding Nrf2 and HIF-1α. All four genes were overexpressed on the day after isolation (day 1). A highly statistically significant difference in expression on day 1 compared to that on day 0 was observed only in the cells of fasted animals; the genes for Srxn1, SOD2, and HIF-1α were significantly overexpressed, while the Nrf2 gene was not (Figure 4a–c). About 5 to 10 times more mRNA of *Srxn1* and *Sod2* was produced compared to that of *Nrf2*. The expression of *Hif1α* was about 10 times lower than that of *Nrf2*. *Sod2* and *Srxn1* were the most expressed genes in the cells of fasted animals on day 1 among both types of cells and all the tested conditions, indicating the induction of strong antioxidant protection during PACOS. Therefore, a highly significant overexpression of antioxidant genes was induced only by fasting.

### 2.3. Stress Response and Modulation of PACOS

We then assessed the gene expression of proteins indicating the general stress response: growth arrest and DNA damage-inducible 45-α, -β, and -γ (Gadd45-α, -β, -γ); ER stress: binding immunoglobulin protein (BiP/Hspa5) and C/EBP homologous protein (Chop); DNA damage response: protein p21, tumour protein 53 (p53); and inflammation: intercellular adhesion molecule 1 (Icam1). Among the Gadd genes, *Gadd45α* was the most overexpressed, particularly in the cells of fasted animals on day 1 (Figure 4e); the largest, statistically significant, difference was compared to the expression in the cells of fasted animals on day 0. The transcription of *Gadd45α* was higher in the cells of the ad libitum-fed animals on the isolation day, so *Gadd45α*’s expression in these cells was not significantly different from that on day 1. *Gadd45β* was also highly expressed in the cells of fasted animals on day 1 and statistically significantly different from the expression levels in the cells of the ad libitum-fed animals on the same day (Figure 4f). *Gadd45γ* was equally overexpressed in both types of cells on both days (Figure 4g).

The genes for the ER chaperone BiP and Chop, which are important in the unfolded protein response, as well as those for the DNA damage response proteins p21 and p53, were significantly more expressed on day 1 than on day 0, with statistically significant differences in expression of all but *Bip*, for the same type of cells (from fasted or fed animals; Figure 4h–k). The expression of *Bip* did not statistically significantly differ between the days in the ad libitum-fed groups. Both *Bip* and *Chop* were significantly upregulated in the cells of fasted animals on day 1. The gene for Icam, a marker of inflammation, was highly expressed in all cells except those from fasted animals on the day of isolation (Figure 4l), implying a possible reduction in inflammatory signalling, at least immediately after fasting. In conclusion, the expression of stress-related genes was higher in the cells of fasted animals 1 day after fasting, supporting the specific stress-response-derived pro-survival effects of fasting.

**Figure 5:**
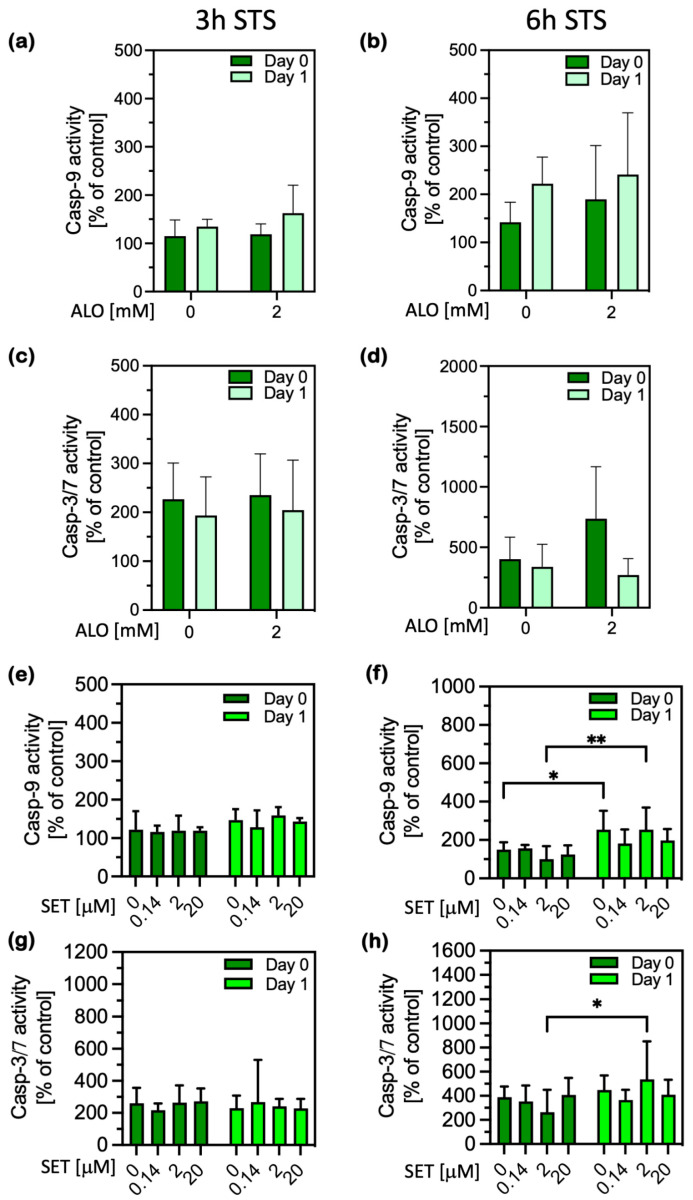
The role of the inhibition of H_2_O_2_ production by xanthine oxidase and NADPH oxidases 1 and 4 in the caspase activation induced by STS in the hepatocytes of fasted animals collected at 0 (day 0, dark green bars) and 24 h (day 1, light green bars) after cell isolation. Caspase activities are expressed as percentages of a nontreated control. (**a**–**d**) Primary hepatocytes were treated with allopurinol (ALO) after isolation, as indicated, before being treated with STS for (**a**,**c**) 3 h (*n* = 5) and (**b**,**d**) 6 h (*n* = 4), as indicated. (**e**–**h**) Primary hepatocytes were treated with the NOX1 and 4 inhibitor setanaxib (SET) after isolation, as indicated, before being treated with STS for 3 h (**e**,**g**) or 6 h (**f**,**h**), as indicated. Data are presented as the mean ± standard deviation (SD) and were analyzed with two-way ANOVA, followed by Tukey’s test. * *p* ≤ 0.05, ** *p* ≤ 0.01. *n* = 4. For the exact *p* values, please refer to Table S1.

To investigate whether H_2_O_2_ was the main messenger conveying the fasting signal to activate the beneficial PACOS, we investigated whether the inhibition of its production by xanthine oxidase and NADPH oxidase (NOX) would reduce or abolish the effects of fasting. When the cells of fasted animals were treated with allopurinol (ALO), an inhibitor of xanthine oxidase, the reduced cellular H_2_O_2_ production did not consistently enable caspase-9 activation upon STS treatment (Figure 5a,b). Nevertheless, caspase-9 may have been activated in at least some of the ALO-treated cells on the isolation day after 6 h of STS treatment (Figure 5b). The inhibition of xanthine oxidase-produced H_2_O_2_ with ALO in the cells of fasted animals did not consistently increase caspase activation, as observed in the cells of fed animals.

Likewise, the inhibition of endogenous H_2_O_2_ production in the cells of fasting animals by treatment with setanaxib (an inhibitor of NOX1 and 4) after cell isolation did not statistically significantly increase the STS-induced caspase-9 activation (Figure 5e,f). Similarly, there was no statistically significant increase in caspase-3 activation (Figure 5g,h). Statistically significant differences were observed for some treatments because of somewhat greater caspase activity on day 1 compared to on the isolation day. Therefore, we did not successfully prevent the stress response phenotype by modulating either xanthine oxidase or two of the NOX enzymes. There are possibly more important sources of H_2_O_2_ production, and/or we did not sufficiently inhibit H_2_O_2_ production by blocking these enzymes only after isolation, as the H_2_O_2_ concentration can also rise substantially during the isolation procedure, i.e., during the liver perfusion. The incomplete, varied inhibition is also supported by the high variability in the caspase activities among the biological replicates. In conclusion, lowering endogenous H_2_O_2_ production after isolating the cells from fasted animals did not abolish the PACOS, at least not by inhibiting a single enzyme at a time, such as xanthine oxidase or NOX 1/4.

**Figure 6:**
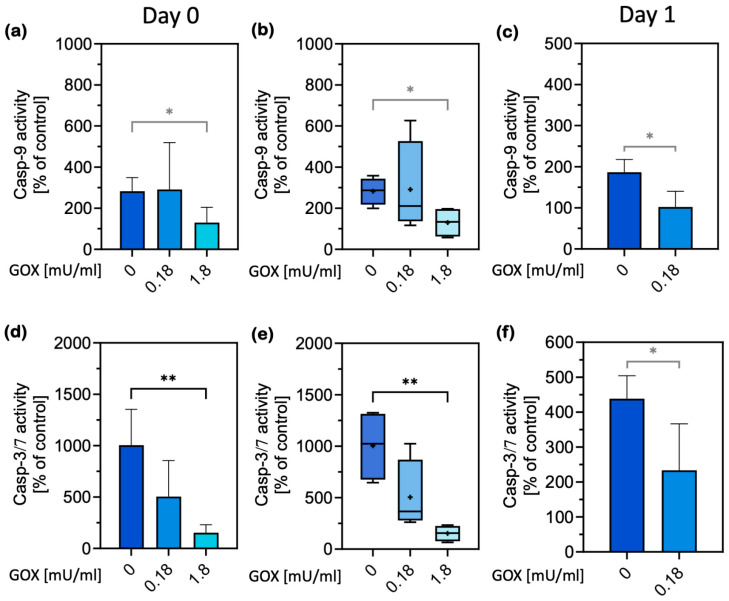
Induction of stress response phenotype in normal hepatocytes by H_2_O_2_ production. H_2_O_2_ production by glucose oxidase (GOX) during the first 6 h after the isolation of hepatocytes prevented caspase-9 activation by STS in the cells of fed animals, inducing the PACOS phenotype. (**a**,**b**,**d**,**e**): Caspase activities on the isolation day, day 0; (**c**,**f**): caspase activities a day after hepatocyte isolation, day 1, expressed as percentages of a nontreated control. Data are presented as the mean ± standard deviation (**a**,**c**,**d**,**f**), and median and interquartile range (**b**,**e**), and were analyzed by one-way ANOVA, followed by Tukey’s test ((**a**,**b**,**d**,**e**); the results are depicted in black) and an unpaired *t*-test ((**c**,**f**) and the comparison of 1.8 mU/mL GOX to 0 in (**a**,**b**)); the results of the unpaired *t*-test are depicted in grey. +: arithmetic mean; * *p* ≤ 0.05, ** *p* ≤ 0.01. *n* = 4. For the exact *p* values, please refer to Table S1.

Next, we increased H_2_O_2_ production in the medium of the cells from ad libitum-fed animals, which showed no stress response, by adding the enzyme glucose oxidase (GOX) to induce PACOS. This prevented caspase-9 activation in a dose-dependent manner (Figure 6a–c). Exogenous H_2_O_2_ produced by GOX (Supplementary Figure S1b) resulted in significant caspase-9 inhibition on both the isolation day and the day after, which was also reflected in the lower activation of caspase-3/7 at all times (Figure 6d–f).

GOX significantly decreased STS-induced caspase-9 activation at a concentration of 1.8 mU/mL on day 0 (Figure 6a,b) and at 0.18 mU/mL on day 1 (Figure 6c), thus mimicking the fasting-induced stress response in the normal cells. This was reflected in statistically significant and dose-dependent reductions in caspase-3 activation (Figure 6d–f). Therefore, we successfully triggered the transformation of non-stress-adapted (normal) primary hepatocytes to the stress response phenotype.

## 3. Discussion

Here, we report that 17 h of fasting in rats results in an apoptosis-attenuating stress response in their liver cells, hepatocytes, that prevents caspase-9 and reduces caspase-3/7 activation upon STS treatment, which typically triggers apoptosis. In contrast, the same trigger highly activates caspases-9 and -3/7 in the hepatocytes of ad libitum-fed animals, which retain the normal phenotype (with no stress response). The decreased ability of moderate stressors to trigger apoptosis was previously attributed to the preapoptotic cell stress response, PACOS, a hormetic response that can prevent cells from undergoing unnecessary apoptosis upon exposure to a subsequent moderate stressor (Supplementary Figure S2) [25,26].

A fasting episode neither disrupts hepatocytes’ cell membranes nor increases apoptosis. The gene encoding the Bcl-2 protein was the least expressed, consistent with a minor role in preventing apoptosis in these cells [27,28]. The genes *BclxL*, *Mcl1*, and *Bax*, which are expressed many times more strongly than other pro- or anti-apoptotic genes, are strongly expressed in the cells of the fasted animals on day 1. The pro-apoptotic *Bax* and the anti-apoptotic *BclxL* are equally overexpressed in the cells of fasted and fed animals. In contrast, the anti-apoptotic *Mcl1* is even more strongly upregulated in the cells of fasted animals. Therefore, an upregulation of anti-apoptotic members, especially Mcl-1 in the cells of fasted animals one day after isolation, can compensate for the overexpression of proapoptotic Bax and Diablo and reflects enhanced survival ability during the stress response. This is consistent with previous findings that Bcl-xL and Mcl-1 are necessary for maintaining hepatocyte integrity [27] and that their protein levels peak 24 h after isolation [26].

The importance of H_2_O_2_ signalling for PACOS initiation was shown before [26] and was confirmed in this study, with larger H_2_O_2_ production and lower activity of antioxidant enzymes (CAT, SOD) in the cells of fasting animals compared to in those of ad libitum-fed animals. This reflects the crucial role of H_2_O_2_ in stress response signalling. The antioxidant protection remained adequate even in the cells of fasted animals, as SOD activity was the lowest and the upregulation of the *Sod2* gene was the highest in these cells one day after isolation. This *Sod2* upregulation may also contribute to improved resilience during the PACOS to subsequent moderate stressors. In addition, fasting increased the expression of genes for other protective proteins, Srxn1 and BiP.

The proteins Gadd45-α, -β, and -γ are cellular stress sensors that, depending on stress and interactions with other proteins, participate in cell cycle arrest, apoptosis, and cell survival [29]. Of the three tested *Gadd45* genes, *Gadd45β* was highly expressed only in the cells of fasted animals one day after isolation. This agrees with previous reports that fasting preferentially promotes *Gadd45β* expression and that the Gadd45-β protein limits the uptake of fatty acids by fatty acid binding protein 1 (FABP1) in hepatocytes [30]. Gadd45-β thus participates in normal lipid metabolism. Its dysregulation has been reported in type 2 diabetic patients, liver steatosis, and obesity [30,31].

According to the expression of the inflammatory marker *Icam1*, there was reduced inflammatory signalling at least immediately after fasting, but only in the cells of fasted animals. This agrees with fasting-associated reductions in the activity of inflammatory pathways in the mouse liver [32] and the results of human studies showing that asthma patients had significantly reduced oxidative stress and inflammatory markers after 2 months of alternate-day fasting [33].

The stress adaptation was triggered by the animal fasting, i.e., the day before cell isolation. The result is a stress response in the cells seen on the day of isolation (day 0) and adaptations/compensations in gene expression observed on day 1. The oxidative modification of enzyme activity occurred faster than the adaptations and compensations by modified gene expression. Additionally, only the cells of the fasted animals maintained maximum urea synthesis for at least another 24 h, indicating undiminished function, in our study. The liver is central for coordinating fasting–feeding transitions and regulating the whole body’s energy and macronutrient metabolism, among other functions: storing, producing, and partitioning glucose; packaging excess lipids for storage and secretion and also oxidising lipids; producing and secreting the majority of the proteins in the blood; controlling the systemic exposure of amino acids entering via the gastrointestinal tract; protein processing for energy; and disposing of nitrogenous waste from the whole organism’s protein degradation [34]. Hepatocytes are the liver’s main metabolic cells. Therefore, it is conceivable that increasing hepatocytes’ resilience maintains and/or improves the metabolic status of the whole body.

Redox signalling is important for triggering and regulating stress responses [21,26,35], with H_2_O_2_ being the main redox sensing, signalling, and regulatory molecule, acting as an oxidant of low-pKa cysteine residues in redox-sensitive proteins [13]. We have confirmed the role of H_2_O_2_ in the initiation of the stress response. There is a small statistically significant increase of H_2_O_2_ production, combined with a significant decrease of SOD and CAT activities on the isolation day in the cells of fasted animals, confirming that there are more ROS in the cells of fasting animals compared to ad-libitum-fed animals on the cell isolation day. The role of H_2_O_2_ as the second messenger was further confirmed by the successful transition of isolated cells between the normal and stress response states upon exposure to the H_2_O_2_ by the H_2_O_2_-producing enzyme GOX.

We did not succeed in the transformation of cells showing the stress response into the normal phenotype by inhibiting either xanthine oxidase or NOX1/4. Although we cannot rule out the possibility that these enzymes were not completely inhibited by ALO and setanaxib, respectively, it is likely that we did not sufficiently block intracellular H_2_O_2_ production. The main sources of ROS in hepatocytes are the mitochondrial electron transport chain and NOX [36,37]. In addition to NOX1 and 4, there are NOX2, DUOX1, and DUOX2 [38], and in addition to xanthine oxidase, there are other H_2_O_2_-producing oxidases, e.g., monoamine oxidases and D-amino acid oxidase [39]. Therefore, the incomplete inhibition of H_2_O_2_ production due to the many sources of H_2_O_2_ in hepatocytes is a more likely explanation for our results.

The addition of the H_2_O_2_-producing enzyme glucose oxidase inhibited STS-induced caspase-9 activation, thus transforming the cells from a normal phenotype to a stress response phenotype. This phenotype is otherwise achieved by overnight fasting. Therefore, we have discovered an underlying mechanism by which acute fasting increases hepatocytes’ resilience and thus possibly contributes to the improvement in metabolic status observed under TRF.

The underlying mechanism by which a single TRF triggers hepatocytes’ adaptive stress response, as described here, could form the basis for devising methods to increase the resilience of the liver. The notion that the PACOS is a beneficial stress response is based on reduced apoptosis and the observed fasting-specific upregulation of protective genes encoding antioxidants (*Srxn1* and *Sod2*), chaperones (*Bip*), and proteins that improve fatty acid metabolism (*Gadd45β*).

The results reported here are the first characterization of a mechanism underlying the effects of fasting. They form the basis for the development of methods to increase the resilience of cells and open up the possibility of searching for fasting mimetics. These research findings also form the basis for the development of clinical applications to increase the resilience of grafts and to improve hepatocyte fitness under acute stress conditions, with potential applications to liver diseases, such as metabolic dysfunction-associated steatohepatitis (MASH), hepatocellular carcinoma, and metabolic disorders such as diabetes and dyslipidaemia, for which intermittent fasting has been reported as a promising intervention [40,41]. As the current work was carried out on a rat model, the data need to be confirmed in humans. We have also shown that fasting animals can modulate their isolated cells. The stress response induced by fasting needs to be taken into account in the accurate interpretation of data from animal and cell research in order to understand the effects of acute overnight fasting, which is used in many laboratory protocols.

## 4. Materials and Methods

Reagents were from Sigma-Aldrich (Merck, Darmstadt, Germany) unless otherwise indicated.

### 4.1. Cell Culture Treatments and Viability Assays

**Figure 7:**
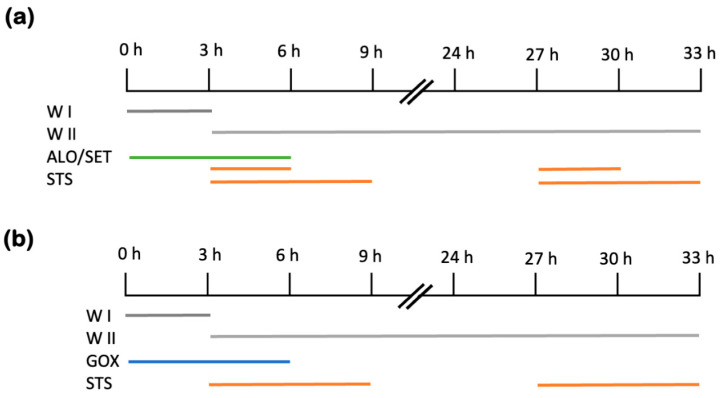
Schematic representation of the cell treatment. All experiments were performed with at least 3 biological replicates. The timeline shows the time from the completion of cell isolation (time 0) and the coloured lines indicate the duration of the respective treatment, either on day 0 (0–9 h) or day 1 (24–33 h), as described in the results. Staurosporine (STS) was always added once, either on day 0 or on day 1, for 3 or 6 h. Treatments with (**a**) alopurinol (ALO), setanaxib (SET), and (**b**) glucose oxidase (GOX) was always performed immediately after isolation. The substances were added to WI medium for 3 h and to WII medium for 3 h.

Primary hepatocytes were isolated from adult male Wistar Hannover rats (200–280 g), as previously described [26,42]. The isolation procedures complied with ethical codes U34401-44/2014/8 and U34401-21/2020/4, granted by the Slovenian Administration for Food Safety, Veterinary Sector, and Plant Protection. Pairs of rats were either fasted for 17 h before cell isolation through chow withdrawal in the cages, with bedding and constant access to water. The animals kept on an ad libitum diet were allowed continuous access to food. The primary hepatocytes were isolated using a reverse two-step perfusion method with collagenase, and the viability of the hepatocytes was confirmed to be at least 90%. For culturing, cells were plated onto collagen-type-I-coated surfaces at 10^5^ cells/cm^2^. Depending on the experimental requirements, different containers were used: 35 mm diameter Petri dishes for urea production analysis, T25 flasks for assessing antioxidant enzyme activities, and 96-well plates for measuring hydrogen peroxide generation. Initially, hepatocytes were incubated under 95% relative humidity and 5% CO_2_ at 37 °C for 3 h. The medium used in this period was Williams E, enriched with 10% fetal bovine serum, antibiotics (penicillin and streptomycin at 100 U/mL), and insulin (0.1 U/mL) (called WI). Then, the Williams E medium was adjusted with 0.03% bovine serum albumin, penicillin and streptomycin (50 U/mL), insulin (0.1 U/mL), and 1 μM hydrocortisone hemisuccinate (called WII). Additionally, the hepatocytes of fasted rats were treated with the xanthine oxidase inhibitor ALO (A8003, Sigma-Aldrich) [43] and NOX inhibitor SET (GKT137831, Cayman Chemical, Ann Arbor, MI, USA) [44] for 6 h on the day of isolation, as depicted in Figure 7.

The hepatocytes of ad libitum-fed rats were treated with GOX (G7141, Sigma-Aldrich) after isolation [45]. This is an enzyme that produces H_2_O_2_ by oxidation of β-D-glucose to D-glucono-δ-lactone in the presence of molecular oxygen [46]. The indicated amount of GOX was added immediately after isolation for 6 h and 3 h, followed by STS caspase activation for 6 h. The cells were harvested 9 h post-isolation on day 0. The cells harvested 27 h after isolation, on day 1, were treated with 1.8 mU/mL GOX for 6 h on the isolation day, as described above, and then, the medium was replaced with WII medium (Figure 7). STS was added 6 h before the end of the experiment.

Cell integrity was assessed by measuring LDH release into the culture supernatants using an LDH Cytotoxicity Detection Kit (MK401, Takara Bio, Kusatsu, Japan) according to the manufacturer’s instructions. For comparisons between the samples, the data were normalized to the total cellular protein, corrected for reaction times (minutes), and expressed as the percentages of LDH released from the cells of fed animals. The MTT assay (Supplementary Figure S1a), which measures the activity of dehydrogenases, was initiated by the 2-h accumulation of 0.5 mg/mL thiazolyl blue tetrazolium bromide (MTT, M5655) dissolved in the cell growth medium. The accumulated crystals were released by DMSO and the absorbance was measured at 550 nm (PerkinElmer, Victor spectrophotometer 1420-050, Waltham, MA, USA).

### 4.2. Protein, H_2_O_2_ and Urea Concentrations

The protein concentrations in the samples were measured using the BCA Protein Assay Kit and the Pierce 660 nm Protein Assay (Pierce, Thermo Scientific, Rockford, IL, USA) for the subsequent analysis of various parameters, including the levels of caspases 3/7 and 9, the level of catalase, the glutathione peroxidase and superoxide dismutase activities, hydrogen peroxide production, glutathione levels, and urea synthesis.

The generation of H_2_O_2_ was quantified using the Amplex Red Hydrogen Peroxide/Peroxidase Assay Kit, following the manufacturer’s instructions (Molecular Probes, Eugene, OR, USA). Measurements were carried out immediately after cell isolation and on the first day post-isolation. The detection of the generated H_2_O_2_ involved measuring the shift in absorbance resulting from the interaction between the released H_2_O_2_ and the Amplex Red reagent.

Urea production, an indicator of hepatocyte functionality and mitochondrial integrity [47,48], was measured after 3 mM ammonium chloride was added to cells in phenol-red-free Williams medium E, at 15 min and 24 h after isolation on days 0 and 1, respectively. The medium was collected every 30 min, up to 2 h, after which the urea concentration was determined as described by Castell and Gomez-Lechon [48].

Total protein synthesis was measured using the puromycin incorporation assay [49]. On the first day of culture, cells seeded in six-well plates were incubated with 1 µM puromycin (Gibco™, A1113803, Thermo Fisher Scientific, Waltham, MA, USA) for 30 min. Following incubation, the cells were washed twice with ice-cold PBS, harvested, and subjected to immunoblotting with anti-puromycin antibody (EMD Millipore MABE343, Merck, Darmstadt, Germany). The quantification of the signal intensity was performed with Image Studio software v.5.2.5. (LI-COR, Lincoln, NE, USA).

### 4.3. Antioxidant Enzymes and Caspase Activity Measurements

The activities of SOD and GPx were measured with assay kits from Cayman Chemical, following the manufacturer’s instructions. The values obtained for enzyme activities were normalized to the protein content in milligrams. The CAT activity was determined by measuring the decrease in H_2_O_2_, which CAT enzymatically converts into oxygen and water [50], following Maehly and Chance’s [51] protocol.

To measure caspase activation, primary hepatocytes were treated for 3 or 6 h with 1 µM STS, which triggers apoptosis through the mitochondrial pathway [52]. STS treatment began 15 min post-isolation on the isolation day (day 0). The activities of caspases-9 and -3/7 were measured with Caspase-Glo 9 and Caspase-Glo 3/7 assays, respectively, following the protocols provided by Promega (Madison, WI, USA).

### 4.4. RNA Isolation and Reverse-Transcription–Quantitative Polymerase Chain Reaction Analysis (RT-qPCR)

The gene expression of growth arrest and DNA damage-inducible 45-α, -β, and -γ was assessed to measure the general stress response; BiP/Hspa5 and Chop for ER stress; protein p21 and tumour protein p53 for DNA damage response; and Icam1 for inflammation [53,54]. The RNA isolation, reverse transcription, and qPCR method were performed as described by Kramar and colleagues [54]. Briefly, duplicate PCRs (≤100 ng cDNA/reaction) were quantified using the QuantStudio 3 Real-Time PCR System with Design and Analysis software 2.5.0 (Thermo Fisher Scientific), which was also used to set the baseline and determine the cycle threshold (Ct). The gene expression was calculated relative to the expression of the 18S rRNA (*Rn18s*) reference gene: target/reference = (E(reference)^Ct(reference)^)/(E(target)^Ct(target)^). TaqMan probes were labelled with the FAM dye (Thermo Fisher Scientific): *Rn18s* (Rn03928990_g1), *Cas9* (#Rn00581212_m1), *Cas3* (#Rn00563902_m1), *Bax* (#Rn01509178_m1), *Bid* (#Rn01459517_m1), *Diablo* (#Rn01480487_g1), *Bcl2* (#Rn99999125_m1), *BclXL* (#Rn00437783_m1), *Mcl1* (#Rn00821024_g1), *Srxn1* (Rn04337926_g1), *Sod2* (Rn00690588_g1), Nrf2/*Nfe2l2* (Rn00582415_m1), *Hif1a* (Rn01472831_m1), *Gadd45α* (Rn00577049_m1), *Gadd45β* (Rn01452530_g1), *Gadd45γ* (Rn01352550_g1), *Bip/Hspa5* (Rn00565250_m1), *Chop/Ddit3* (Rn00492098_g1), *p21* (Rn00589996_m1), *Tp53* (Rn00755717_m1), and *Icam1* (Rn00564227_m1).

### 4.5. Statistical Analysis

Statistics were calculated using GraphPad Prism 9.0.0 and 10.2.3, with an in-built algorithm that tests the equality of variances from medians, using the Brown–Forsythe test. Either two-way or one-way ANOVA (the latter only in Figure 6) was used for comparisons between two independent variables, followed by Tukey’s multiple-comparison test to compare every mean with every other mean. Student’s *t*-test was additionally used to measure the difference between two samples from hungry and fed animals. The data are presented as the mean ± SD (unless otherwise stated); the number of biological replicates (*n*) are noted in the figure legends and the exact *p* values are provided in Tables S1 and S2. The significance level was set at *p* ≤ 0.05.

## Data Availability

Data will be available from the repository of the University of Ljubljana upon the manuscript’s publication.

## Supplementary Materials

The following supporting information can be downloaded at: www.mdpi.com/article/10.3390/ijms26030999/s1.
