# Supplementary material for: Short-Term Fasting Induces Hepatocytes’ Stress Response and Increases Their Resilience
Source: Int J Mol Sci. 2025 Jan 24;26(3):999. doi: 10.3390/ijms26030999 (PMC11817670; doi:10.3390/ijms26030999)
Supplement: Supplementary file 1 [file ijms-26-00999-s001.zip › Figures S1 and S2.pdf]

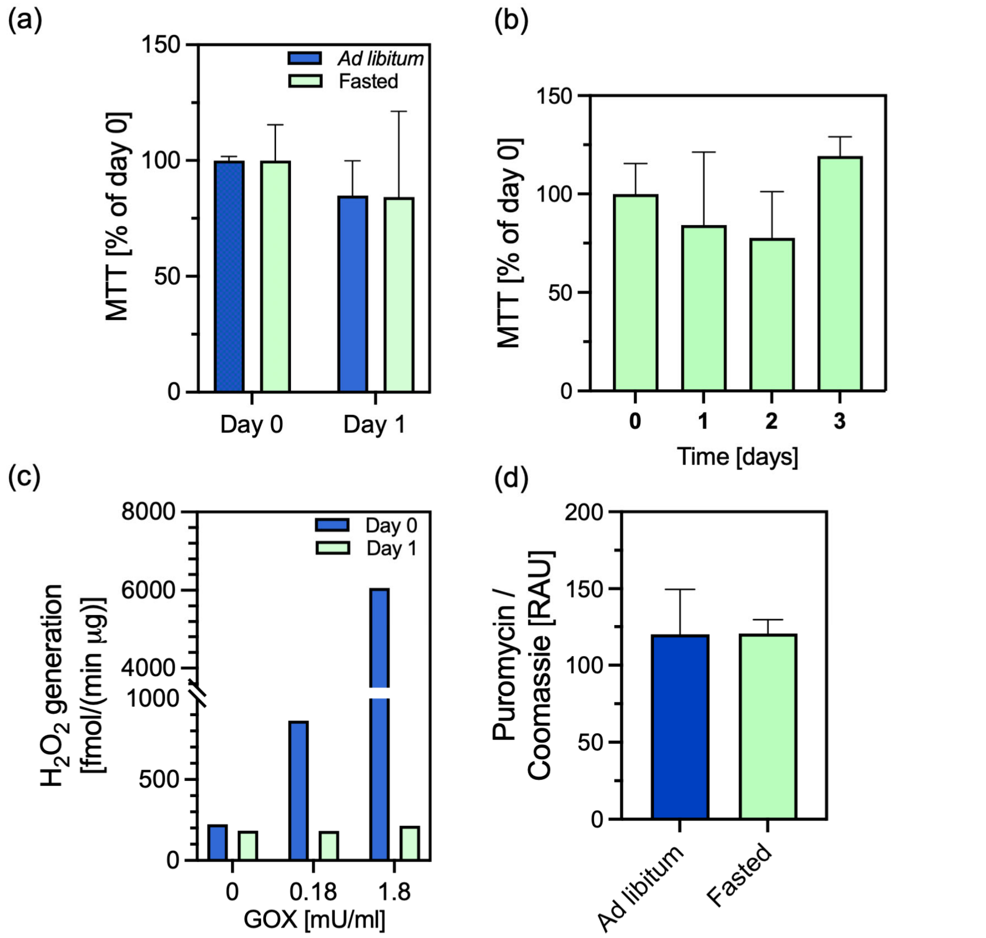

Figure S1. (**a**) Survival of cells from fed and fasted animals, MTT assay. (**b**) Survival of cells from fasted animals, MTT assay. (**c**) Generation of H_2O_2 upon GOX treatment. GOX was always added only on day 0 for 6 hours, as shown in Figure 7b. (**d**) Protein synthesis in the cells of fed and fasted animals. RAU: relative arbitrary units.

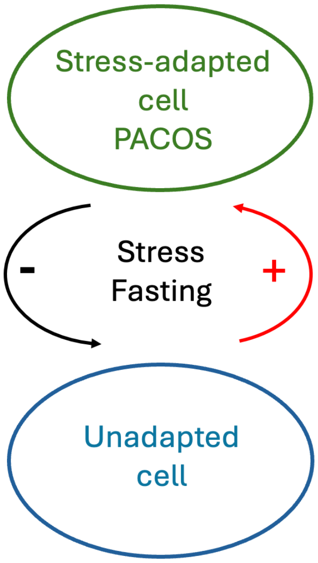

Figure S2. Flowchart summarizing a transition from a normal to a stress-adapted cell (PACOS).
