# Supplementary material for: Short-Term Fasting Induces Hepatocytes’ Stress Response and Increases Their Resilience
Source: Int J Mol Sci. 2025 Jan 24;26(3):999. doi: 10.3390/ijms26030999 (PMC11817670; doi:10.3390/ijms26030999)
Supplement: Supplementary file 1 [file ijms-26-00999-s001.zip › Table S1.pdf]

Table S1. Statistics results. n.s: not significant; NA: not available.

	Two-way ANOVA - Tukey's			
	Ad libitum Day 0 vs Day 1	**Fasted** Day 0 vs Day 1	**Day 0** *Ad libitum* vs Fasted	**Day 1** *Ad libitum* vs Fasted
**Fig**	**p-value**	**p-value**	**p-value**	**p-value**
**1a**	n.s.	n.s.	n.s.	n.s.
**1b**	0.0061	n.s.	n.s.	0.0260
**1c**	n.s.	0.0231	0.0324	n.s.
**1d**	n.s.	0.0173	0.0007	n.s.
**1e**	n.s.	0.0495	n.s.	n.s.
**1f**	n.s.	n.s.	0.0146	n.s.

	Two-way ANOVA - Tukey's			
	Ad libitum Day 0 vs Day 1	**Fasted** Day 0 vs Day 1	**Day 0** *Ad libitum* vs Fasted	**Day 1** *Ad libitum* vs Fasted
**Fig**	**p-value**	**p-value**	**p-value**	**p-value**
**3a**	0.0149	0.0009	n.s.	n.s.
**3b**	0.0165	0.0288	n.s.	n.s.
**3c**	n.s.	n.s.	n.s.	n.s.
**3d**	0.0039	0.0006	0.0266	0.0032

	t-test
	**Day 0** Fasted vs *Ad libitum*
**Fig**	**p-value**
**3a**	0.0261
**3c**	0.0122

	Two-way ANOVA - Tukey's									
	**0 mM** Day 0 vs Day 1	**2 mM** Day 0 vs Day 1	**Day 0** 0 vs 2 mM	**Day 1** 0 vs 2 mM						
**Fig**	**p-value**	**p-value**	**p-value**	**p-value**						
**5a**	n.s.	n.s.	n.s.	n.s.						
**5b**	n.s.	n.s.	n.s.	n.s.						
**5c**	n.s.	n.s.	n.s.	n.s.						
**5d**	n.s.	n.s.	n.s.	n.s.						
	**0 µM** Day 0 vs Day 1	**0.14 µM** Day 0 vs Day 1	**2 µM** Day 0 vs Day 1	**20 µM** Day 0 vs Day 1	**Day 0** 0 vs 0.14 µM	**Day 0** 0 vs 2 µM	**Day 0** 0 vs 20 µM	**Day 1** 0 vs 0.14 µM	**Day 1** 0 vs 2 µM	**Day 1** 0 vs 20 µM
	**p-value**	**p-value**	**p-value**	**p-value**	**p-value**	**p-value**	**p-value**	**p-value**	**p-value**	**p-value**
**5e**	n.s.	n.s.	n.s.	n.s.	n.s.	n.s.	n.s.	n.s.	n.s.	n.s.
**5f**	0.0500	n.s.	0.0054	n.s.	n.s.	n.s.	n.s.	n.s.	n.s.	n.s.
**5g**	n.s.	n.s.	n.s.	n.s.	n.s.	n.s.	n.s.	n.s.	n.s.	n.s.
**5h**	n.s.	n.s.	0.0271	n.s.	n.s.	n.s.	n.s.	n.s.	n.s.	n.s.

	t-test	
	**0 vs 0.18**	**0 vs 1.8**
**Fig**	**p-value**	**p-value**
**6a**	n.s.	0.0215
**6b**	n.s.	0.0215
**6c**	0.0138	NA
**6d**	n.s.	0.0031
**6e**	n.s.	0.0031
**6f**	0.0327	NA

	One-way ANOVA - Tukey's	
	**0 vs 0.18**	**0 vs 1.8**
**Fig**	**p-value**	**p-value**
**6a**	n.s.	n.s.
**6b**	n.s.	n.s.
**6d**	n.s.	0.0062
**6e**	n.s.	0.0062
