# Supplementary material for: Short-Term Fasting Induces Hepatocytes’ Stress Response and Increases Their Resilience
Source: Int J Mol Sci. 2025 Jan 24;26(3):999. doi: 10.3390/ijms26030999 (PMC11817670; doi:10.3390/ijms26030999)
Supplement: Supplementary file 1 [file ijms-26-00999-s001.zip › Table S2.pdf]

Table S2. Statistical results for gene expression experiments. n.s: not significant.

		**Two-way ANOVA - Tukey's test**			
		Ad libitum Day 0 vs Day 1	**Fasted** Day 0 vs Day 1	**Day 0** *Ad libitum* vs Fasted	**Day 1** *Ad libitum* vs Fasted
**Fig**	**Gene**	**p-value**	**p-value**	**p-value**	**p-value**
**2a**	*Casp-9*	**0.0157**	**0.0004**	n.s.	n.s.
**2b**	*Casp-3*	n.s.	n.s.	n.s.	n.s.
**2c**	*Bax*	**0.0162**	**0.0038**	n.s.	n.s.
**2d**	*Bid*	n.s.	**0.0018**	n.s.	n.s.
**2e**	*Diablo*	**0.0385**	**0.0002**	n.s.	**0.0111**
**2f**	*Bcl2*	n.s.	**0.0092**	n.s.	n.s.
**2g**	*Bclxl*	n.s.	**0.0007**	n.s.	n.s.
**2h**	*Mcl1*	n.s.	**0.0001**	n.s.	**0.0013**
**4a**	*Srxn1*	n.s.	**0.0059**	n.s.	n.s.
**4b**	*Sod2*	n.s.	**0.0046**	n.s.	n.s.
**4c**	*Nfe2l2*	n.s.	n.s.	n.s.	n.s.
**4d**	*Hif1α*	n.s.	**0.0042**	n.s.	**0.0021**
**4e**	*Gadd45α*	n.s.	**0.0003**	n.s.	n.s.
**4f**	*Gadd45β*	n.s.	n.s.	n.s.	**0.0169**
**4g**	*Gadd45γ*	n.s.	n.s.	n.s.	n.s.
**4h**	*Bip*	n.s.	**<0.0001**	n.s.	**0.0002**
**4i**	*Chop*	**0.0198**	**<0.0001**	n.s.	**0.0112**
**4j**	*p21*	**0.0179**	**<0.0001**	n.s.	n.s.
**4k**	*Tp53*	**0.0500**	**0.0004**	n.s.	n.s.
**4l**	*Icam1*	n.s.	**0.0115**	n.s.	n.s.
